# Exploring the role of IL-1β in inflammatory bowel disease pathogenesis

**DOI:** 10.3389/fmed.2024.1307394

**Published:** 2024-01-22

**Authors:** Ioanna Aggeletopoulou, Maria Kalafateli, Efthymios P. Tsounis, Christos Triantos

**Affiliations:** ^1^Division of Gastroenterology, Department of Internal Medicine, University Hospital of Patras, Patras, Greece; ^2^Department of Gastroenterology, General Hospital of Patras, Patras, Greece

**Keywords:** dysbiosis, inflammasome, inflammatory bowel disease, interleukin 1β, IL-1R, intestinal inflammation, miRNAs

## Abstract

Interleukin 1β (IL-1β) is a significant mediator of inflammation and tissue damage in IBD. The balance between IL-1β and its endogenous inhibitor-IL-1Ra-, plays a critical role in both initiation and regulation of inflammation. However, the precise role of IL-1β as a causative factor in IBD or simply a consequence of inflammation remains unclear. This review summarizes current knowledge on the molecular and cellular characteristics of IL-1β, describes the existing evidence on the role of this cytokine as a modulator of intestinal homeostasis and an activator of inflammatory responses, and also discusses the role of microRNAs in the regulation of IL-1β-related inflammatory responses in IBD. Current evidence indicates that IL-1β is involved in several aspects during IBD as it greatly contributes to the induction of pro-inflammatory responses through the recruitment and activation of immune cells to the gut mucosa. In parallel, IL-1β is involved in the intestinal barrier disruption and modulates the differentiation and function of T helper (Th) cells by activating the Th17 cell differentiation, known to be involved in the pathogenesis of IBD. Dysbiosis in the gut can also stimulate immune cells to release IL-1β, which, in turn, promotes inflammation. Lastly, increasing evidence pinpoints the central role of miRNAs involvement in IL-1β-related signaling during IBD, particularly in the maintenance of homeostasis within the intestinal epithelium. In conclusion, given the crucial role of IL-1β in the promotion of inflammation and immune responses in IBD, the targeting of this cytokine or its receptors represents a promising therapeutic approach. Further research into the IL-1β-associated post-transcriptional modifications may elucidate the intricate role of this cytokine in immunomodulation.

## 1 Introduction

Inflammatory bowel diseases (IBD), mainly ulcerative colitis (UC) and Crohn’s disease (CD), are chronic inflammatory conditions of the gastrointestinal tract (1). These conditions are marked by persistent mucosal inflammation caused by both adaptive and innate uncontrolled immune responses. UC presents widespread inflammation and ulcers that can extend along a varying distance from the rectum to the caecum. On the other hand, CD is mainly characterized by transmural inflammation occurring at any location in the gastrointestinal tract, with the terminal ileum and colon being the most affected areas. The global incidence and prevalence of IBD have risen significantly over the past decade in both Western and Eastern countries, highlighting a significant public health challenge (2–4). Consequently, there is a great need for the development of novel treatment approaches.

Apart from their clinicoparhological presentation on the gastrointestinal tract, IBD frequently lead to extra-intestinal disease. These complications derive from the IBD-induced persistent and systemic inflammatory state, disrupting various signaling pathways and altering the expression of regulatory mediators like cytokines and microRNAs (miRNAs) (5, 6). The exact cause of IBD is still not fully understood, but recent advances have shed light on the underlying processes which drive these diseases. The pathophysiology and development of IBD involve several mechanisms, including dysregulated immune responses, environmental factors, alteration in gut microorganisms (dysbiosis), and genetic changes related to the disease (7–13).

**Figure 1:**
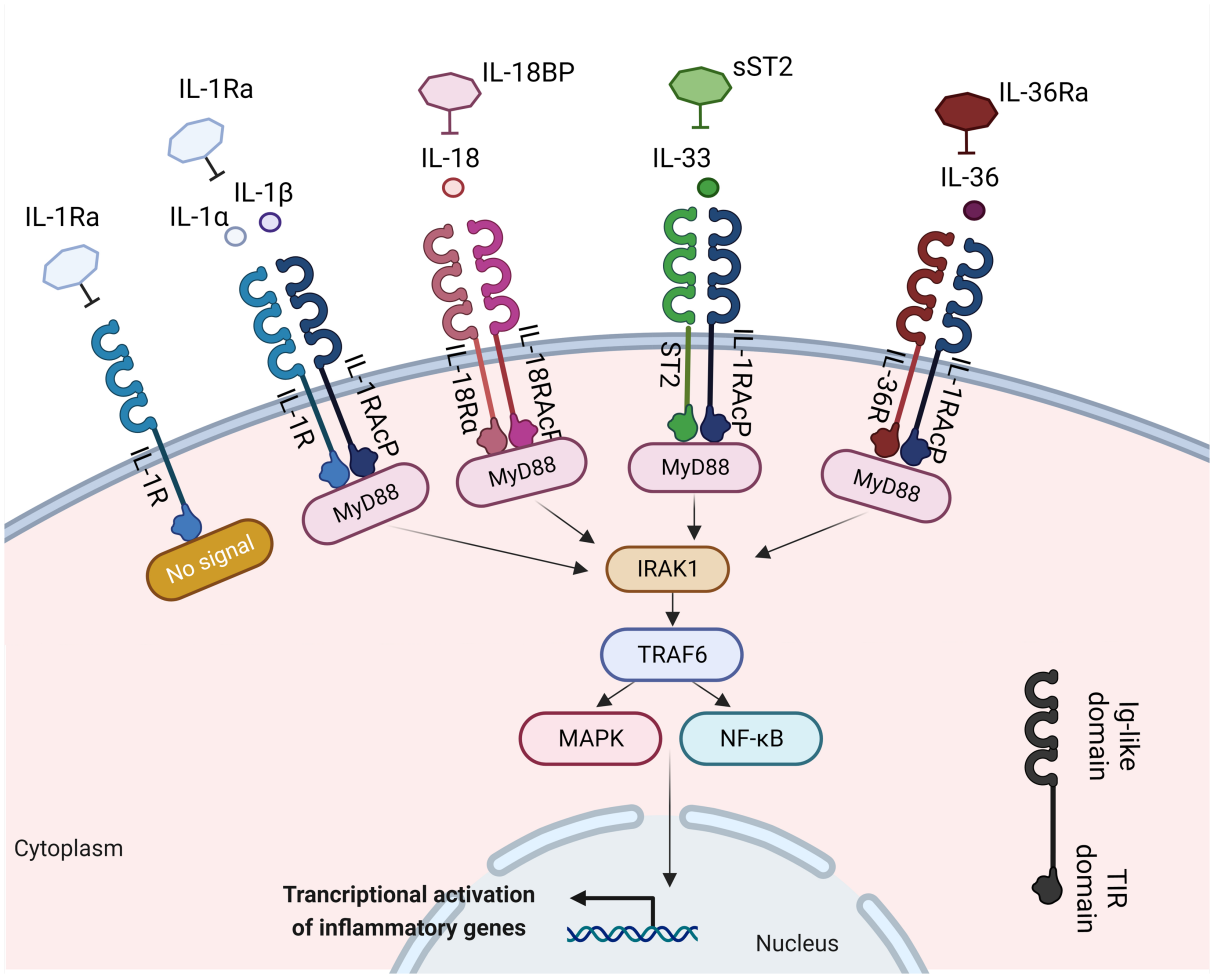
IL-1 family members with inflammatory function, their cognate receptors, and their antagonists. Interleukin-1α (IL-1α), IL-1β, IL-18, IL-33, and IL-36 bind to their receptors within the IL-1R family. This binding facilitates the recruitment of myeloid differentiation primary response 88 (MyD-88), interleukin-1 receptor-associated kinase 1 (IRAK1), and TNF receptor associated factor 6 (TRAF6). This process results into the activation of nuclear factor kappa-light-chain-enhancer of activated B cells (NF-κB) and mitogen-activated protein kinase (MAPK) signaling pathways, inducing the transcription of various inflammatory genes. Created with BioRender.com. IL-1Ra, interleukin 1 receptor antagonist; IL-1R, interleukin 1 receptor; IL-1, interleukin 1; IL-1RAcP, interleukin-1 receptor accessory protein; MyD88, myeloid differentiation primary response 88; ST2, interleukin 33 receptor; IRAK-1, interleukin-1 receptor-associated kinase 1; TRAF6, TNF receptor associated factor 6; NF-κB, nuclear factor kappa-light-chain-enhancer of activated B cells; MAPK, mitogen-activated protein kinase; Ig, immunoglobulin; TIR, Toll-IL-1-receptor.

The interplay between the innate and adaptive immune responses is highly affected by various cytokines. Any disruption in this communication can lead to the initiation and propagation of inflammatory response in the mucosal tissue. Among these cytokines, the interleukin (IL)-1 plays a crucial role in innate immune responses, being an essential modulator of inflammation in a wide range of human disorders (14). The IL-1 family comprises a group of 11 cytokines, including seven agonistic ligands (IL-1α, IL-1β, IL-18, IL-33, IL-36α, IL-36β, IL-36γ), three antagonist ligands [IL-1 receptor antagonist (IL-1Ra), IL-36Ra, IL-38] which exert their effects through various heterodimeric receptor complexes, and one anti-inflammatory cytokine, the IL-37 (14, 15) (Figure 1). A wide range of immune cells, such as monocytes, dendritic cells (DC), macrophages, natural killer (NK) cells, activated T and B cells, along with non-hematopoietic cells like epithelial cells and keratinocytes, produce IL-1 family cytokines in response to various triggers, including pathogen-and damage-associated molecular patterns (PAMPs and DAMPs, respectively), as well as other cytokines, notably the tumor necrosis factor (TNF) (16).

The IL-1 family of cytokines and receptors has been the subject of extensive research in the context of IBD, due to their widely accepted involvement in the development of various inflammatory disorders. The critical role of IL-1 as a mediator of innate immune response and a promoter of inflammation has been reported in patients with IBD (17). The targeting of the IL-1-related pathways has been explored to alleviate IBD symptoms, but the outcomes have been variable. The aim of the current review was to provide an overview of the molecular and cellular characteristics of IL-1β and to describe the existing evidence on the role of this cytokine as a modulator of intestinal homeostasis and an activator of inflammatory responses, in the course of IBD. Moreover, the most recent findings on the role of miRNAs in the regulation of the IL-1β-related inflammatory responses in experimental IBD will be discussed.

## 2 IL-1 signaling

A structural resemblance has been observed between the IL-1 family receptors and the toll-like receptors (TLRs); thus, these two families are commonly categorized together as the Toll/IL-1 Receptor (TIR) superfamily (18). Each receptor consists of an extracellular ligand-binding domain, an intracellular TIR domain which is responsible for the signal transduction and a transmembrane helix. Moreover, both families employ the myeloid differentiation primary response 88 (MyD88) as an adaptor protein for downstream signaling and employ the IL-1 receptor accessory protein (IL-1RAcP). The specificity for their cognate ligands is determined by sequence residues in the extracellular immunoglobulin-like domain of IL-1 family receptors (19) (Figure 1). The signaling through IL-1Rs is similar to MyD88-dependent TLR signaling, because of the homology between the TLRs and the IL-1R (20).

The IL-1 group can be categorized into three subfamilies based on the length of their precursor proteins and the N-terminal segments: IL-1, IL-18, and IL-36 (21). IL-1 ligands bind to the extracellular regions of their ligand binding receptor chain (22) (Figure 1). The receptor antagonists consist of several ligand binding chains [IL-1R1, IL-33R (ST2), IL-18Rα, and IL-36R]. Following the interaction of the ligand with these chains, they recruit either the IL-1RAcP for IL-1R1, IL-33R, and IL-36R, or the IL-18RAcP chain for the IL-18R, respectively (22). Moreover, signaling molecules like the IL-1R-associated kinases (IRAKs) and the intermediate protein TNF receptor-associated factor 6 (TRAF6), which are also found in many TLR signaling pathways, play a vital role in IL-1 signaling. The dimerization event is crucial for the transmission of the IL-1 signal downstream (23). Upon activation, the receptor complexes induce the activation of pro-inflammatory intracellular signaling mediated by [mitogen-activated protein (MAP) kinases] MAPK-and NF-κB- (nuclear factor kappa B) dependent pathways and greatly contribute to the development of both adaptive and innate inflammation (24). IL-1R-mediated signaling results in the transmission of multiple signals, many of which can regulate both local and systemic immune responses. IL-1β plays a crucial role not only in chronic and acute inflammation (25, 26) but also in the induction of adhesion molecules expression on endothelial cells, promoting the maturation of DCs which regulate the recruitment and stimulation of lymphocytes and NK cells, and act as an endogenous pyrogen capable of causing fever (16).

Beyond the classical pro-inflammatory functions of IL-1 family, several cytokines of this family influence the downstream effector T cell responses, by guiding their differentiation into various pathways. In particular, IL-1β drives the T helper 17 (Th17) cells differentiation, while IL-18 and IL-33 promote the expansion of Th1 and Th2 cells, respectively (27, 28). Moreover, the IL-1 family cytokines can also induce the secretion of cytokines from diverse immune cells, such as innate lymphoid cells (ILCs) and intraepithelial lymphocytes (IELs), which constitutively express receptors for this family of cytokines. IL-1β induces robust IL-17 production from both γδ T cells and type 3 ILCs (ILC3s), whereas IL-33 and IL-18 can trigger the secretion of type 1 or type 2 cytokines from ILC1s and ILC2s, respectively (29).

Due to their pro-inflammatory nature and the diverse array of cells expressing IL-1 family receptors, the functions of IL-1 family cytokines are firmly regulated. Except for IL-1Ra, all IL-1 family cytokines are first produced as biologically inactive precursors and necessitate post-translational modifications before the activation of their respective receptors. The maturation of IL-1β, IL-18, and IL-37 involves the cleavage by inflammatory caspases; post-translational cleavage commonly occur in IL-1α, IL-33, and IL-36 subfamilies (30). The IL-1β pro-form is functionally inert and is not able to bind to IL-1RI (31). The enzyme responsible for the cleavage of pro-IL-1β was initially identified as IL-1β converting enzyme (ICE) and was later renamed caspase-1; this enzyme is a component of a multi-protein complex called inflammasome (32). IL-1β does not display intracellular functionality and is secreted from the cell in response to specific stimuli (33). In parallel, the active cytokines are intrinsically modulated by a network of antagonists and/or decoy receptors which are able to inhibit pro-inflammatory signal transduction by IL-1 family members (34).

The IL-1 family is commonly related to pro-inflammatory responses; in particular, IL-1β has been identified as a critical mediator of inflammation-induced pathology in various autoimmune and auto-inflammatory disorders (17, 35–38). Thus, the signaling pathways related to these cytokines constitute potential targets for various conditions including IBD (39).

## 3 IL-1β and NLRP3 inflammasome

The NLRP3 inflammasome is a key component of the immune system response to infection, tissue damage, and cellular stress (40, 41). It plays a crucial role on the regulation of inflammation by controlling the activation of pro-inflammatory cytokines, particularly IL-1β and IL-18 (42).

**Figure 2:**
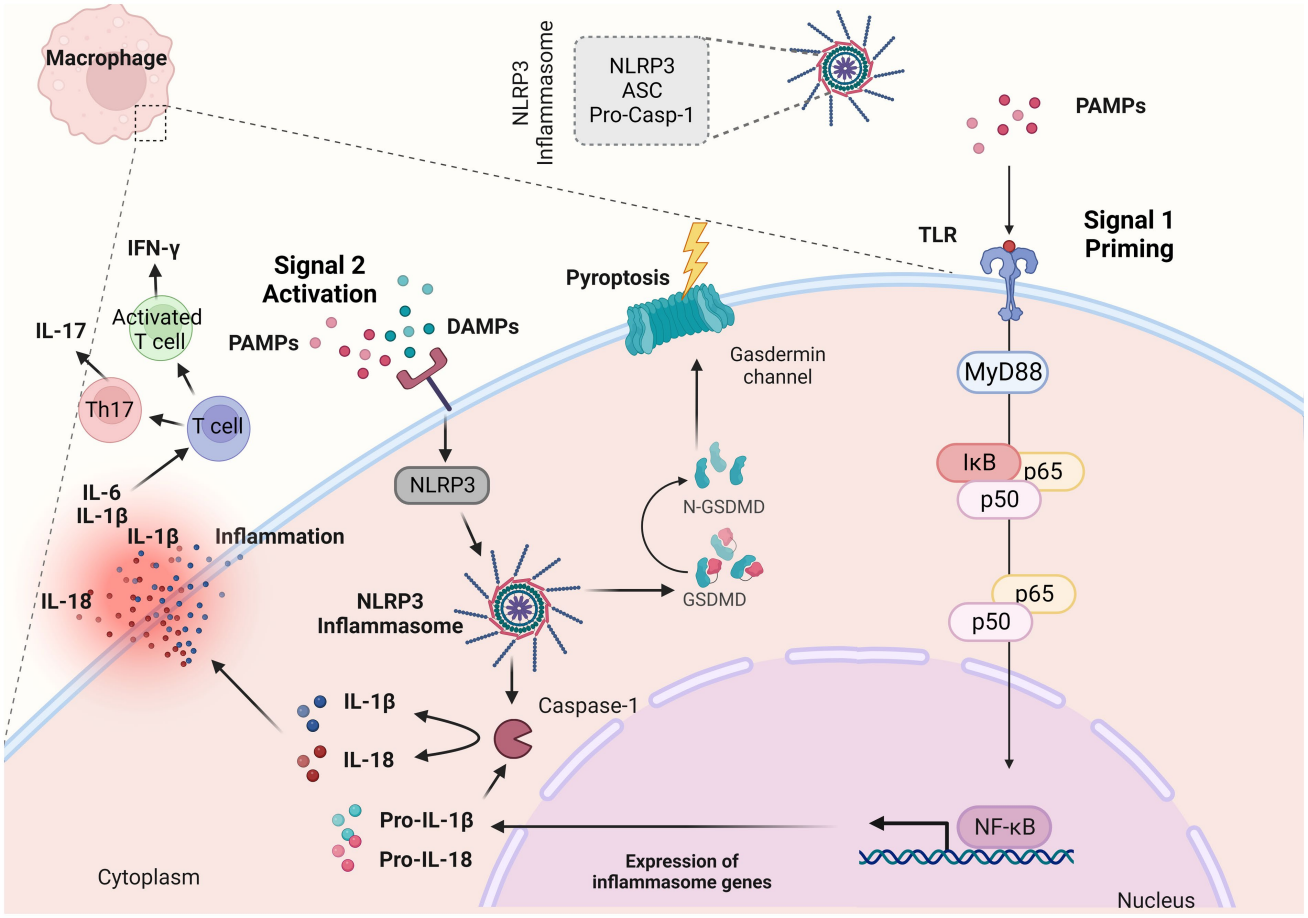
NLRP3 inflammasome complex and IL-1β release. The NLRP3 inflammasome is a multiprotein complex that assembles in response to “danger signals” which can include pathogen-associated molecular patterns (PAMPs) and damage-associated molecular patterns (DAMPs). These signals are recognized by various receptors, including toll-like receptors and NOD-like receptors (NLRs). When activated, NLRP3 recruits other proteins to form the inflammasome complex. This complex then activates the caspase-1, an enzyme that cleaves pro-interleukin 1β (IL-1β) and pro-IL-18 into their active forms (IL-1β and IL-18). During an inflammatory response, IL-1β along with other proinflammatory cytokines such as tumor necrosis factor (TNF-α) and IL-6 orchestrate the inflammatory response within the lamina propria. Created with BioRender.com. ASC, adaptor-apoptosis-associated speck-like protein; pro-casp-1, pro-caspase; PAMPs, pathogen-associated molecular patterns; DAMPs, damage-associated molecular patterns; TLRs, toll-like receptors; GSDMD, Gasdermin D; IL-1β, interleukin 1β; IL-18, interleukin 18; NF-κB, nuclear factor kappa-light-chain-enhancer of activated B cells; MyD88, myeloid differentiation primary response 88; IκBα, inhibitor of kappa B alpha; IFN-γ, interferon γ; Th17, T helper 17.

The NLRP3 inflammasome, which is present within mucosal macrophages and dendritic cells, serves as the cellular machinery responsible for the production of the mature (cleaved) variant of IL-1β that is subsequently released by these cells (42–45). The NLRP3 is comprised of three main components, the sensor NLRP3 protein, the adaptor-apoptosis-associated speck-like protein (ASC) and the effector protein-caspase-1 (46, 47) (Figure 2). The activation of NLRP3 inflammasome consists of two signals: a) signal 1 (priming), which is promoted by pathogen recognition receptors (PRRs), such as TLRs stimulated by PAMPs (42–45) (Figure 2); resulting into the transcriptional activation of inflammasome genes including Nlrp3, IL-1β and IL-18 by transcription factors, such as NF-κB (48) and b) signal 2 (activation) which starts with the binding of PAMPs or DAMPs in specific membrane receptors, activating various signaling events and leading to the activation of NLRP3, oligomerization, and generation of NLRP3 inflammasome complex (48) (Figure 2). The NLRP3 inflammasome activation results into the cleavage of Gasdermin D and activation of pyroptosis and/or the formation of the active caspase-1 which is responsible for the cleavage of pro-IL-1β and pro-IL-18 into their biologically active forms, IL-1β and IL-18 (46–48) (Figure 2).

## 4 Il-1β and NLRP3 inflammasome in IBD

The dysregulation of the NLRP3 inflammasome has been implicated in various inflammatory and autoimmune diseases, including IBD (49). IL-1β promotes inflammation by attracting immune cells to the site of infection or injury but also enhances the immune response by influencing the activation and differentiation of immune cells. In the context of infection, this can be beneficial for clearing pathogens (50, 51). However, when the inflammasome is chronically activated or dysregulated, it can contribute to excessive inflammation and tissue damage, as seen in IBD. Inflammasome activation in intestinal cells can lead to increased production of IL-1β and IL-18, driving inflammation and tissue damage in the gut (50, 51). During the inflammatory response caused by colitis-induced model or IBD, IL-1β along with other proinflammatory cytokines such as TNF-α and IL-6 orchestrate the inflammatory response within the lamina propria (52, 53). IL-1β along with IL-6, induce the differentiation of naïve T cells into Th17 cells, which produce IL-17, or T cells that produce interferon γ (IFN-γ) (54). These cells are responsible for the development and maintenance of inflammation.

In experimental animal studies, administration of IL-1β or IL-18 minimized colitis progression in NLRP3 knockout (KO) mice; however there was no effect in colitis severity in wild type (WT) mice (55). Targeting and blockade of IL-1β by genetic and pharmacological procedures in colitis mice resulted in attenuated colitis (56). Seo et al. (57) showed that inflammatory monocytes and monocyte-derived IL-1β are essential in driving pathology, as IL-1β KO mice and CCR2 KO mice were found to be protected from acute DSS-colitis.

In human studies, data have shown that NLRP3 inflammasome expression was stimulated *ex vivo* in CD patients with a concomitant increase of IL-1β levels in peripheral blood mononuclear cells (PBMCs) *in vitro*; on the other hand, activation of the NLRP3 was reported in late disease stage of disease in UC patients (58). Another study demonstrated an increase of NLRP3 components (NLRP3, IL-1β, ASC and caspase-1) in colonic biopsies of both UC and CD patients, which was positively related to disease activity (59).

## 5 Il-1β in IBD

Most data on IBD research have focused on IL-1β, which has been found increased in these disorders (60–63) (Table 1). In particular, IL-1β expression was higher in plasma and colonic mucosa tissue of patients with IBD (60–63, 67). Augmented IL-1β production from macrophages and colonic tissue in patients with IBD has been associated with disease severity and chronic intestinal inflammation (59). During intestinal inflammatory conditions, a significant increase in IL-1β levels, and a positive association between the mucosal inflammation severity and the IL-1β levels have been reported (64). Mononuclear cells, mainly macrophages within the lamina propria, are the primary source of the increased IL-1β levels in inflamed tissue biopsies from patients with active IBD (65). The extent of this elevation has been correlated to the overall severity of the disease (65). The majority of cells in the intestinal mucosa express IL-1Rs, allowing them to respond to IL-1 activation. Thus, any alterations in the levels of IL-1 could potentially influence the various cell populations, including lymphoid, myeloid, and non-hematopoietic lineages, leading to important alterations in the immunological profile of the gastrointestinal tract. Zhou et al. (66), demonstrated that constitutive signals of intestinal macrophage-derived IL-1β, produced in response to commensal stimuli, critically contribute to the production of T regulatory cells (Tregs) which are responsible for the maintenance of immune equilibrium under physiological conditions. This process was found to rely on IL-1β-responsive ILC3s, which drive the Tregs differentiation by the secretion of IL-2 (66). When IL-1 signaling was specifically ablated in ILC3s, the induction of Tregs and oral tolerance to dietary antigens were abrogated (66). Intriguingly, decreased secretion of IL-2 by ILC3s was observed in patients with active CD, indicating that this pathway might be clinically important and could be targeted as a therapeutic option (66).

**Table 1:** Synopsis of studies related to the role of IL-1β οn IBD.

Study (reference)	Year	Journal	Material used	Study outcomes
McAlindon et al. (60)	1998	Gut	IBD colons	IBD colonic macrophages ➔ synthesis of both precursor & mature IL-1β IBD colon-derived macrophages ➔ production of active (p20) ICE ICE inhibitor ➔ significant decreases the mature IL-1β release by isolated IBD macrophages
Reimund et al. (61)	1996	J Clin Immunol	Inflamed mucosa from IBD patients	IBD mucosa-derived organ cultures ➔ increased production of IL-1β *vs* healthy mucosa
Mahida et al. (62)	1989	Gut	Mucosa from IBD patients	Increased IL-1β release by mononuclear cells from inflamed IBD mucosa *vs* normal colonic mucosa LPS stimulation ➔ induction of IL-1β production by mononuclear cells from active IBD mucosa
Reinecker et al. (63)	1993	Clin Exp Immunol	LPMNC isolated from colonic IBD biopsies	IBD-derived LPMNC ➔ spontaneous production of IL-1β
Ransoan et al. (59)	2018	Int J Mol Sci	Paired UC & CD biopsies	In active UC & CD ➔ upregulated IL-1β & IL-1β localization in the infiltrate of lamina propria immune cells In non-inflammatory state ➔ IL-1β localization in the near-exclusive epithelial cell layer
Al-Sadi et al. (64)	2008	J Immunol	Caco-2 cell culture	IL-1β ➔ progressive increase in MLCK expression Linear correlation between increase of IL-1β-induced MLCK expression & increase in Caco-2 TJ permeability Suppression of the IL-1β-induced increase in MLCK protein expression & activity ➔ protection from increase in Caco-2 TJ permeability MLCK protein knock-down ➔ prevention of IL-1β-induced increase in Caco-2 TJ permeability IL-1β-induced increase in MLCK mRNA & protein expression & Caco-2 TJ permeability ➔ mediated by NF-kB activation
Jones et al. (65)	2018	Front Immunol	Colonic IBD-derived myeloid cells & murine DSS induced colitis	In murine blood monocytes ➔ increase in IL-1β release In IBD colon ➔ monocytes are the dominant local source of IL-1β & TNF
Zhou et al. (66)	2019	Nature	Experimental model	IL-1β selectively induces IL-2 expression by ILC3s in the small intestine Macrophages in the small intestine produce IL-1β Activation of this pathway involves MYD88- & NOD2-dependent sensing of microbiota

IBD, inflammatory bowel disease; IL-1β, interleukin-1 beta; ICE, IL-1 beta converting enzyme; LPS, lipopolysaccharide; LPMNC, lamina propria mononuclear cells; ILCs, innate lymphoid cells; IL-1R, IL-1 receptor; UC, ulcerative colitis; CD, Crohn disease; MLCK, myosin L chain kinase; TJ, tight junction; NF-Κb, nuclear factor kappa B; TNF, tumor necrosis factor; DSS, dextran sulphate sodium; ILC3s, group-3 innate lymphoid cells; MyD88, myeloid differentiation primary response 88; NOD, nucleotide-binding oligomerization domain.

## 6 IL-1β-targeted therapeutic options

### 6.1 Experimental models

IL-1Ra, a natural antagonist of IL-1RI is the best characterized endogenous modulator of IL-1 bioactivity, commonly secreted simultaneously with IL-1, and aims to suppress IL-1Ra related signaling and to blockade the excessive activation of a pro-inflammatory response. In pre-clinical studies, IL-1Ra KO mice have shown high susceptibility to several autoimmune diseases; the study by Rogier et al. (68), reported that in IL-1Ra KO mice, the arthritis severity was significantly influenced by the composition of the commensal microbiota, indicating a strong microbial component in this condition. In the absence of IL-1Ra, an abnormal type-17 response was developed both in the gut mucosa and systemically, which could be transferred to WT mice through fecal transfer or co-habitation (68). However, this IL-17-mediated auto-inflammation was notably reduced by using antibiotics targeting the IL-17-driving bacteria, suggesting that besides direct antagonism of the IL-1 bioactivity, IL-1Ra also plays a crucial role on the maintenance of a proper microbiota environment in the gastrointestinal tract (68).

Single immunoglobulin IL-1 related-receptor (SIGIRR) is a suppressive receptor of the IL-1 family, also known as TIR8; the extracellular SIGIRR domain can interact with the IL-1RI, resulting to blockade of dimerization with the IL-1RAcP (69). This receptor is essential for the gastrointestinal tract as both intestinal DCs and IECs derived by SIGIRR-deficient mice display an imbalanced immunological profile characterized by a constant increase in inflammatory genes and heightened responsiveness to TLR ligands (70). Furthermore, SIGIRR KO mice have been found more susceptible to intestinal inflammation and carcinogenesis in models of dextran sulfate sodium (DSS)-induced colitis and azoxymethane (AOM)/DSS-induced colorectal cancer (CRC), respectively (71, 72). SIGIRR has also been reported to inhibit Th17 cell proliferation by counteracting IL-1 signaling and glycolytic metabolism, outcome of critical significance in regard to the development of spontaneous colitis observed in IL-10 KO mice, in which Th17 cells greatly contribute to disease pathology (73). Hence, the SIGIRR expression by both hematopoietic and non-hematopoietic cells in the gastrointestinal tract appears to be crucial for the maintenance of immune homeostasis.

Bersudsky et al. (74), reported amelioration of intestinal inflammation upon DSS administration through the neutralization of IL-1α, but not by exogenous administration of rIL-1Ra or anti-IL-1β antibodies.

### 6.2 IL-1β targeted drugs

IL-1β has been closely associated with the failure of anti-TNF therapy (75). IL1Ra can suppress the biological effect of IL-1β, serving as a natural decoy (76). Anakinra (Kineret, Swedish Orphan Biovitrum AB, Stockholm, Sweden) is a recombinant form of the IL-1Ra, that inhibits the activity of IL-1β, and has been primarily used as a treatment for autoimmune diseases that involve dysregulated IL-1 signaling (77, 78). While Anakinra has been studied for its potential in various inflammatory conditions, including IBD, its effectiveness in IBD treatment has been limited and its use is not a standard approach. Clinical trials and studies have explored the use of Anakinra in both CD and UC; however, the evidence supporting its use in IBD is not as strong as for other available therapies (79). In an experimental study using Winnie-TNF-KO mice, the effect of recombinant Anakinra has been evaluated (38). The results showed reduction of the histologic score in the distal colon and decreased levels of IFN-γ–expressing CD8+ T cells in the lamina propria and mesenteric lymph node–derived T cells following Anakinra administration, suggesting its use in primary non-responders to anti-TNF treatment (38).

Canakinumab, a human monoclonal antibody targeting IL-1β which has been approved for the treatment of various autoinflammatory conditions (80, 81), has been also explored in the context of IBD. A recent study assessed how patients with very early onset IBD, exhibiting a phenotype resembling that of monogenic autoinflammatory diseases, responded clinically to canakinumab (82). The study suggested the use of canakinumab for children with very early onset IBD or older pediatric and adult IBD patients with refractory autoinflammatory phenotype, highlighting its potential use as an alternative to other treatment options because of the restricted immunosuppression and the limited number of side effects (82).

## 7 Il-1β role in IBD pathogenesis

Although increased levels of IL-1β have been observed in the intestinal tissue of patients with IBD, there is scarce evidence on the IL-1β contribution to intestinal pathology (Table 2). Coccia et al. (54), examined the role of IL-1β in driving innate and adaptive gut pathology. The results showed that IL-1β promoted innate pathology in *Helicobacter hepaticus*-induced intestinal inflammation through the increased recruitment of granulocytes and ILCs and the activation of ILCs (54). A critical contribution of IL-1R signal in the recruitment and survival of pathogenic Th cells in the colonic mucosa, has also been reported (54). IL-1β has been found to induce Th17 responses from Th cells and ILCs in the intestinal tissue, while a synergistic interplay between IL-1β and IL-23 signaling for the maintenance of both innate and adaptive responses has also been demonstrated (54). Another study indicated a major role of IL-1 in IBD pathogenesis by examining the histological alterations of IL-1Ra KO mice in the small intestine (17). A significant decrease in the villus heights of small intestine and in the villus width in the ileum has been observed, accompanied by an increase in goblet cell number and mucin secretion (17). Moreover, IL-1α and IL-1β immunopositivity and infiltration of polymorphonuclear cells and macrophages were higher, whereas IL-1R1 expression and expression of tight and adhesion junctions was reduced in IL-1Ra KO mice compared to WT mice (17). Within the heterogeneous tissular inflammatory responses, a recent study identified co-expressed gene modules in patients with IBD, which mapped to specific histopathological and cellular characteristics (pathotypes) (83). High neutrophil infiltration, fibroblasts stimulation and vascular remodeling at the regions of deep ulceration were observed (83). In the ulcer bed, the induced fibroblasts exhibited IL-1R-dependent neutrophil-chemoattractant features (83). These pathotype-related neutrophil and fibroblast properties were induced in non-responders to various treatments, suggesting a biological rationale for the blockade of IL-1 signaling in ulcerating disease (83).

**Table 2:** Synopsis of studies related to the role of IL-1β οn IBD pathology.

Study (reference)	Year	Journal	Material used	Study outcomes
Coccia et al. (54)	2012	J Exp Med	Experimental models of chronic intestinal inflammation	IL-1β induces innate pathology in *Helicobacter hepaticus*-associated intestinal inflammation by increasing the recruitment of granulocytes & ILCs’ accumulation & stimulation T cell transfer colitis model ➔ critical role for T cell-specific IL-1R signals in the accumulation & survival of pathogenic Th cells in the colon IL-1β induces Th17 responses from Th cells & ILCs in the intestine IL-1β & IL-23 ➔ interaction to maintain intestinal innate & adaptive inflammatory responses
Dosh et al. (17)	2019	Oncotarget	IL-1rn−/− BALB/c experimental model	Small intestine of BALB/c IL-1rn−/− mice ➔ reduced villus height Ileum of BALB/c IL-1rn−/− mice ➔ reduced villus height & villi width & increased goblet cell number & mucin production *vs* WT mice IL-1rn−/− mice ➔ increased IL-1α & IL-1β immunopositivity but decreased IL-1R1 expression IL-1rn−/− mice ➔ increased polymorphonuclear & macrophage infiltration but dramatically decreased expression of tight & adhesion junctions
Friedrich et al. (83)	2021	Nat Med	IBD patients	Co-expressed gene modules within the heterogeneous tissular inflammatory response which map to specific histopathological & cellular characteristics (pathotypes) Pathotype definition ➔ high neutrophil infiltration, fibroblasts activation & vascular remodeling at regions of deep ulceration Activated fibroblasts in the ulcer bed present IL-1R-dependent neutrophil-chemoattractant effects Pathotype-related neutrophil & fibroblast signatures ➔ increased in non-responders to various treatment options suggesting a biological rationale for the blockade of IL-1 signaling in ulcerating disease

IL-1β, interleukin-1 beta; ILCs, innate lymphoid cells; Th17, T helper 17 cells; WT, wild type; IBD, inflammatory bowel disease.

## 8 miRNAs as regulators of IL-1β signaling in IBD

**Figure 3:**
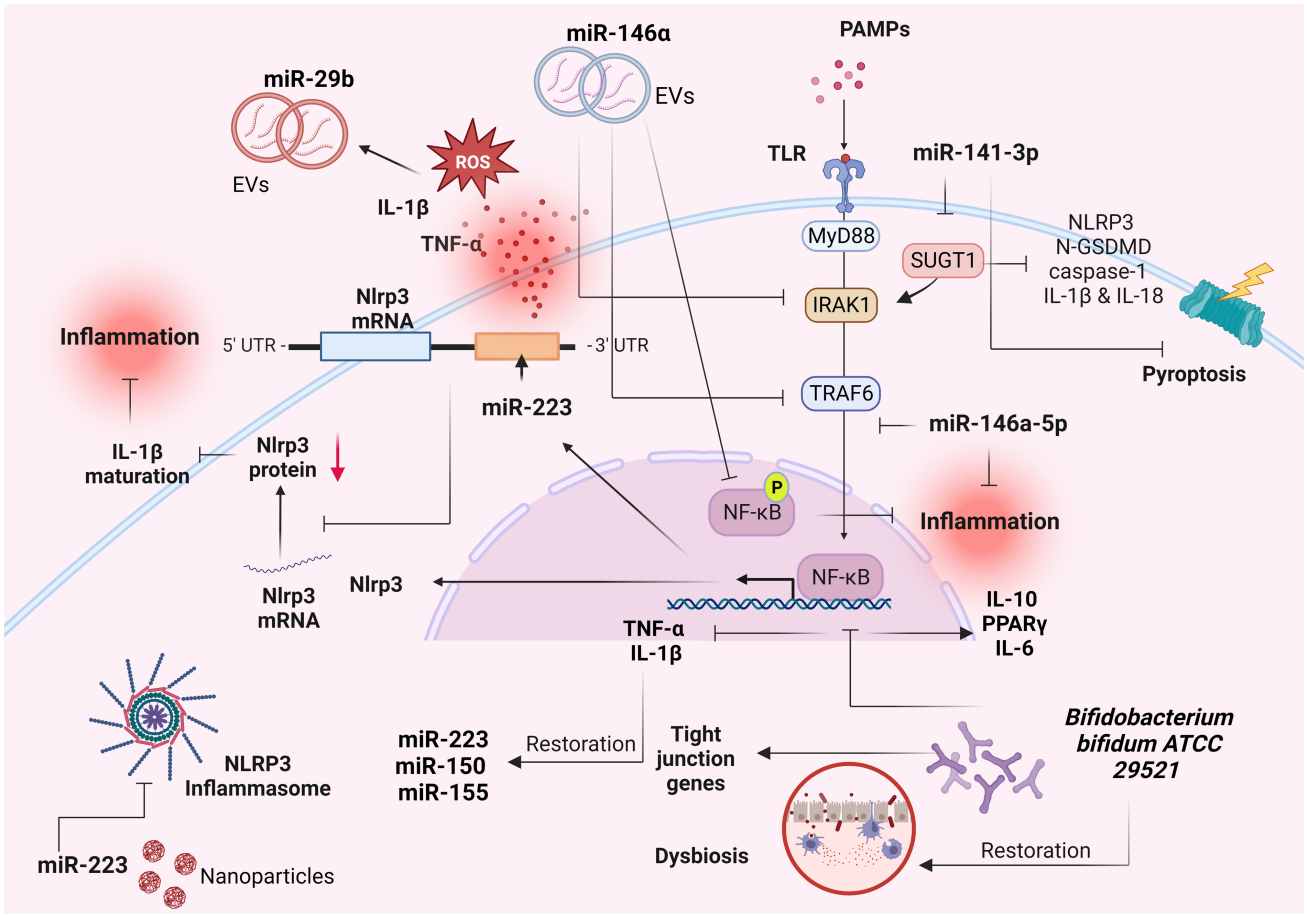
Role of miRNAs as IL-1β-related signaling regulators in IBD. Several miRNAs have been implicated in the pathogenesis of inflammatory bowel diseases (IBD) and in particular with the IL-1β-related signaling. These molecules constitute potential biomarkers for IBD diagnosis and prognosis. Created with BioRender.com. miR-, miRNA; IL-1β, interleukin 1β; TNF-a, tumor necrosis factor a; ROS, reactive oxygen species; UTR, untranslated region; EVs, extracellular vesicles; PAMPs, pathogen-associated molecular patterns; TLRs, toll-like receptors; MyD88, myeloid differentiation primary response 88; IRAK-1, interleukin-1 receptor-associated kinase 1; TRAF6, TNF receptor associated factor 6; NF-κB, nuclear factor kappa-light-chain-enhancer of activated B cells; p, phosphorylation; GSDMD, Gasdermin D; PPARγ, peroxisome proliferator activated receptor gamma.

Numerous miRNAs have emerged as pivotal modulators of mucosal barrier function and inflammatory response, within the mucosa (84, 85). MiRNAs constitute a group of endogenous non-coding RNAs which modulate various cellular activities within eukaryotes by selectively targeting the 3′-untranslated region of mRNAs, leading to mRNA degradation or translational suppression (84, 86). Consequently, miRNAs assume essential regulatory roles across diverse pathways and biological processes, including among others, autoimmunity, inflammation, and the maintenance of immunological equilibrium (87–89). Several studies have pinpointed the central involvement of miRNAs in the pathological advancement of IBD, particularly in the maintenance of homeostasis within the intestinal epithelium (90–92) (Figure 3).

Neudecker et al. (93), revealed a miR-223–NLRP3–IL-1β regulatory circuit, presenting a miR-223-mediated suppression of the intestinal inflammation by the blockade of the NLRP3 inflammasome (Figure 3). Induced NLRP3 expression combined with increased IL-1β levels were found as the predominant features for the initiation of colitis in miR-223 KO mice (93). Exhaustion of CCR2+ inflammatory monocytes and blockade of IL-1β or NLRP3 resulted into phenotype abrogation (93).

MiR-146a-5p, a significant anti-inflammatory miRNA, has been found to effectively inhibit TLR4-mediated NF-κB-regulated gene expression and to operate as a counteractive feedback modulator of the innate immune response by targeting the signaling molecules IRAK1 and TRAF6 (94) (Figure 3). These adapter proteins hold pivotal roles in the signaling pathways downstream of TNF-α and IL-1β (7). These results were confirmed by a recent study which revealed the mechanism by which the miR-146a-5p negatively modulated the IL-1β-induced inflammation by the downregulation of the IRAK1/TRAF6 signaling pathway, in human IECs (95). In particular, the expression of miR-146a-5p and inflammatory markers were found increased in IL-1β-activated Caco-2 cells (95). Upregulation of miR-146a-5p resulted in downregulation of inflammatory markers, whereas its downregulation induced their expression (95), suggesting that this process occurs by selected downregulation of IRAK1/TRAF6 signaling (95). Wu et al. (94), showed that extracellular vesicles (EVs) containing miR-146a, significantly suppressed the expression of TRAF6 and IRAK1 in experimental colitis caused by 2,4,6-trinitrobenzenesulfonic acid (TNBS), in rats (Figure 3). In parallel, the phosphorylation of NF-κB p65 and IκBα (inhibitor of NF-κB) was decreased following the administration of over-expressed miR-146a EVs, leading to inhibited secretion of TNF-α, IL-6 and IL-1β and thus ameliorated colitis (94). Administration of nanoparticle with overexpressed miR-223 showed amelioration of experimental colitis, and decrease of NLRP3 levels and IL-1β secretion (93).

In a recent study, miR-141-3p mimic reduced the expression of pyroptosis-related proteins including NLRP3, N-GSDMD, caspase-1 and the production of inflammatory factors IL-1β and IL-18 in colon epithelial cells, by targeting the SUGT chaperone (96) (Figure 3). Moreover, miR-141-3p ameliorated the inflammatory phenotype of mouse colonic mucosal tissue in a DSS-induced colitis model (96). TNFα, IL-1β and H_2_O_2_ significantly induced the expression of miR-29b in CCD841 CoN colon epithelial cells (97). The plasma exosomes secreted by CCD841 CoN cells presented increased levels of miR-29b as well, triggered by TNFα, IL-1β or H_2_O_2_ (97) (Figure 3). These results suggest that the presence of reactive oxygen species and proinflammatory cytokines in the inflamed mucosa of patients with IBD induced the IECs in the intestine to produce miR-29b-overexpresed exosomes, which are secreted into the blood stream (97).

The beneficial role of the probiotic strain *Bifidobacterium bifidum* ATCC 29521 was examined in a DSS-induced colitis model (98). The strain restored the DSS-induced injury by modulating the expression of tight junction proteins and immune markers in the colon, including the NF-κB p65-mediated downregulation of inflammatory genes TNF-α and IL-1β in the *Bifido* mice group (98) (Figure 3). Moreover, the downregulation of TNF-α and IL-1β was related to the restoration of miR-150, miR-155 and miR-223 in the *Bifido* group (98). The restored DSS-induced dysbiosis in the *Bifido* group demonstrated that *B. bifidum* strain exhibited its probiotic role through an anti-inflammatory effect regulated by miRNA-associated proteins and NF-κB modulation (98).

## 9 Conclusions and prospects

The involvement of IL-1 family members in the gastrointestinal tract extends beyond their role in eliciting inflammatory responses, as these cytokines also play a vital role in the maintenance of proper barrier function. The specific impact of the IL-1 family on the gastrointestinal homeostasis or IBD pathogenesis varies depending on factors such as disease state, genetic predisposition due to genetic alterations, influence of gut microbiota composition and existence of natural antagonists.

The NLRP3 inflammasome is highly important for the production of IL-1β and is considered as a critical modulatory tool for the maintenance of intestinal homeostasis. Thus, targeting the NLRP3 inflammasome regulation and the related endpoints including IL-1β during aberrant inflammation in IBD, will pave the way into the development of novel therapeutic approaches.

The stimulation of TLRs induces both innate and adaptive immune responses and activates the NF-κB signaling which is a major regulator of cytokine secretion in inflammatory disorders. Thus, NF-κB blockade may constitute an attractive avenue for the attenuation of chronic intestinal inflammation in IBD. Current research has focused on the inhibition of inflammatory responses through the suppression of NF-κB binding activity by targeting IRAK1 and TRAF6, critical modulators of IL-1 signaling. Accumulating evidence reveals the crucial role of miRNAs in the pathological advancement of IBD, particularly in the regulation of innate immune responses during intestinal inflammation and the maintenance of homeostasis within the intestinal epithelium. The effect of miRNAs in the inhibition of NF-κB activity has shown great potential; however, the long-term impact of miRNAs on NF-κB signaling pathway-associated epithelial homeostasis remains elusive and need further investigation. In depth research into the post-transcriptional modifications which modulate the IL-1β activity, may highlight the relevant and/or differential role of this cytokine in immunomodulation.

Despite the fact that IL-1β represents an appealing target for IBD treatment, a comprehensive understanding of its function in IBD warrants further investigation. This includes the conduction of additional research using animal models featuring cell-specific conditional knockouts along with well-defined procedures to minimize the impact of variations in the commensal microbiota among different strains.

## Author contributions

IA: Conceptualization, Data curation, Investigation, Resources, Visualization, Writing – original draft. MK: Resources, Writing – original draft. ET: Resources, Writing – original draft. CT: Conceptualization, Project administration, Supervision, Writing – review & editing.
